# Resistance to CDK4/6 Inhibitors in Estrogen Receptor-Positive Breast Cancer

**DOI:** 10.3390/ijms222212292

**Published:** 2021-11-14

**Authors:** Erin R. Scheidemann, Ayesha N. Shajahan-Haq

**Affiliations:** Lombardi Comprehensive Cancer Center, Department of Oncology, Georgetown University Medical Center, Washington, DC 20057, USA; ers112@georgetown.edu

**Keywords:** ER+ breast cancer, antiestrogens, CDK4/6 inhibitors, palbociclib, ribociclib, abemaciclib

## Abstract

Estrogen receptor-positive (ER+) breast cancer is the most common form of breast cancer. Antiestrogens were the first therapy aimed at treating this subtype, but resistance to these warranted the development of a new treatment option. CDK4/6 inhibitors address this problem by halting cell cycle progression in ER+ cells, and have proven to be successful in the clinic. Unfortunately, both intrinsic and acquired resistance to CDK4/6 inhibitors are common. Numerous mechanisms of how resistance occurs have been identified to date, including the activation of prominent growth signaling pathways, the loss of tumor-suppressive genes, and noncanonical cell cycle function. Many of these have been successfully targeted and demonstrate the ability to overcome resistance to CDK4/6 inhibitors in preclinical and clinical trials. Future studies should focus on the development of biomarkers so that patients likely to be resistant to CDK4/6 inhibition can initially be given alternative methods of treatment.

## 1. Introduction

Breast cancer is the most common type of malignancy in women. Metastatic breast cancer can result when cancer is not detected early or develops resistance to early-stage treatment. Consequently, breast cancer metastasis is the second leading cause of cancer mortality in women in developed countries [1]. Breast cancers are classified based on the status of the estrogen receptor (ER), progesterone receptor (PR), and human epidermal growth factor receptor (HER2). Of these, ER+ breast cancer is the most common, comprising about 70% of cases [2]. These tumors rely on the binding of estrogen to the ER for proliferation.

The first treatments available for ER+ breast cancer patients were antiestrogens. There are three types of antiestrogens approved for use in hormonal breast cancers. Selective estrogen receptor modulators (SERMs), such as tamoxifen, prevent estrogen from binding to the ER. Selective ER downregulators (SERDs), such as fulvestrant, inhibit ER-mediated signaling. Lastly, aromatase inhibitors (AIs) such as letrozole deplete the cell of estrogen by inhibiting the upstream aromatase enzyme. While these therapies are effective in many ER+ breast cancers, only about 50% of patients respond [3]. The need to overcome this resistance led to the development of cell cycle inhibitors for ER+ breast tumors: CDK4/6 inhibitors. Targeting Cyclin-dependent Kinase 4 (CDK4) and Cyclin-dependent Kinase 6 (CDK6) in ER+ breast cancer is useful because these cancers often have the cyclin D1–CDK4/6–Rb pathway activated. These cytostatic drugs work to stall the cell cycle and prevent further cell division [4].

The cell cycle is the process in which cells duplicate their genetic material and appropriately divide into daughter cells [5]. The cell cycle is organized into four main stages. The first step, G1, is when cells primarily grow. Here, they produce protein and increase in size. Next, in the S phase, DNA synthesis occurs. The cell makes a second copy of its DNA in preparation for division. The G2 phase follows to ensure that the replicated DNA is error-free. Lastly, the M phase is when mitosis occurs. The cell fully segregates its DNA copies and splits into two daughter cells [6].

Checkpoints in the cell cycle function to control each step. At each checkpoint, steps are taken to ensure that DNA integrity is maintained so that proliferation and cell division occur without error. Many abnormal occurrences in the cell can trigger a checkpoint to prevent cell cycle progression [5]. Cells use size as an indication of progression, especially at the G1 and G2 steps, because a larger size is indicative of more material within the cell. If the cell has already duplicated its DNA, it will be larger and can progress to the next phase. Nutrient status is also used to determine the ability of cells to move onto the next phase. If ample nutrients are not available, the cell cycle will stall [5].

DNA damage is another reason that the cell cycle must be halted. DNA replication is an imperfect process and can result in mutations being present on the newly synthesized strand of DNA. The cell needs time to repair the damage before proceeding onto the next phase. If this repair does not occur, mutations can accumulate and be passed to daughter cells, eventually resulting in the synthesis of unintended proteins. This can have detrimental effects on the cell or on the organism as a whole [5]. There are three damage checkpoints throughout the cell cycle in mammals: G1/S, intra-S, and G2/M. Throughout these checkpoints, certain regulator proteins have the ability to sense and attach to the damaged area of the DNA, triggering transducer proteins to signal that damage is present. Cell cycle regulators phosphorylate effector kinases via mediator proteins. Effector kinases then cause cell cycle arrest so that repair can take place [7]. Furthermore, when errors occur during DNA replication, blockades such as modified dNTPs, protein–DNA complexes, or a reduced deoxynucleotide triphosphate (dNTP) pool can cause a stall to occur. These errors induce replication stalling and trigger a cascade of proteins to bind and become activated to stabilize the replisome and prevent progression while repair of the mistake takes place or the dNTP pool is restored [5].

The mitotic spindle checkpoint occurs in metaphase to ensure that the spindle is correctly assembled. If microtubules are incorrectly attached to the chromatids, uneven distribution of the chromatids results. Proper spindle assembly is monitored based on measures of microtubule tension and attachment. Incorrect assembly is sensed by Mitotic Arrest Deficient 2 (Mad2), which inhibits Cdc20 to inactivate the Anaphase Promoting Complex (APC). The inactivation of APC prevents the cell from entering anaphase [8].

While there are many points in the cell cycle during which the cell can sense an abnormality and halt progression, it is not a failsafe system. The cell cycle can become dysregulated in many ways, leading to profound effects on the cell. The dysregulation of cell cycle components can cause the cell to mistakenly continue through division, even if mistakes occurred at any step along the way. Simply put, the accumulation of mutations over the course of multiple divisions can cause a cell to transform and become cancerous [5].

CDK4/6 inhibitors target cells with a dysregulated cell cycle. Using these drugs on cells with overactive CDK4/6 signaling helps to stall progression and prevent further division. While CDK4/6 inhibitors have proven to be highly effective in the clinic for ER+ breast cancer patients, de novo or developed resistance to these drugs is common. Identifying the mechanisms that cause either form of resistance is crucial, as it can allow these anomalies to be targeted. Hopefully, this would cause resistant patients to regain sensitivity to CDK4/6 inhibitors. Eventually, using biomarkers to identify which patients are likely to not respond would allow clinicians to personalize their treatment to prevent the initial onset of resistance.

## 2. CDK4/6 Are Good Targets in ER+ Breast Cancer

One particular cell cycle pathway that is mutated in breast cancer involves CDK4/6. When CDK4/6 are overactive, they cause the cell to erroneously pass through the G1/S checkpoint [9]. During the G1 phase of the cell cycle, cyclin-dependent kinase inhibitor 2A (CDKN2A) and cyclin-dependent kinase inhibitor 2B (CDKN2B) prevent the action of CDK4/6 [9]. To progress in the cell cycle, an upstream signal causes CDKN2A and CDKN2B to dissociate from CDK4/6 [10]. This promotes the binding of cyclin D1 and CDK4/6. The formation of this complex releases ATP, which supplies energy to phosphorylate the retinoblastoma protein (Rb). Rb is a tumor suppressor protein that, when bound to the E2 transcription factor (E2F), prevents cell cycle progression. Once it is activated, the phosphorylated Rb dissociates from the Rb–E2F complex. Once free, E2F can assist in the transcription of target genes, which allows for the G1/S transition to take place [11].

Cyclin dependent kinases (CDKs) are regulated by posttranslational modifications, such as ubiquitination, phosphorylation, and binding of endogenous inhibitors. CDK4/6 signaling can become overactive in cancer cells via genetic or epigenetic mechanisms [12]. When this occurs, cells erroneously transition from G1 to S in the cell cycle. Overactive CDK4 and/or CDK6 can prevent senescence from occurring, which further aids in tumorigenic transformation [12]. Furthermore, this pathway mediates the action of estrogen on proliferation [13]. Consequently, targeting CDK4/6 in ER+ breast cancers is an effective therapeutic strategy.

CDK4/6 inhibitors are particularly useful in ER+ patients because they typically have the cyclin D–CDK4/6–INK4–Rb pathway activated [14]. Because estrogen is mitogenic, and thus leads to increases in both cyclin D1 and CDK4/6 activity, it causes excess proliferation in hormone-regulated breast cancers [3]. Therefore, targeting components of this pathway helps to circumvent resistance to antiestrogens. It is also important that most patients who develop endocrine resistance still have a functioning Rb pathway. This renders the targeting of CDK4/6 useful, especially since it allows Rb to remain functional and helps the cell control its growth [15]. Furthermore, the MutL mismatch repair (MMR) complex is commonly dysregulated in ER+ breast cancer. As a result, CDK4 becomes overactive because checkpoint kinase 2 (CHK2), a kinase responsible for DNA damage repair, can no longer inhibit it [16]. The analysis of data from a clinical trial (NCT01723774) of endocrine-resistant patients has confirmed that inhibiting CDK4/6 is effective in MutL-defective, endocrine-resistant ER+ tumors for this reason [16]. Another way that endocrine resistance can be caused is via Ankyrin Repeat and LEM Domain Containing 2 (LEM4) overexpression. LEM4 stabilizes Rb and CDK4 to promote the phosphorylation of Rb. In turn, this encourages the G1/S transition, even in the presence of tamoxifen [17]. Due to mutations like these, targeting CDK4/6 in ER+ breast cancer effectively elicits a response in endocrine-resistant patients.

## 3. The Three Approved CDK4/6 Inhibitors

There are three FDA-approved inhibitors of CDK4/6 for use in patients with ER+ breast cancer: palbociclib, ribociclib, and abemaciclib. Palbociclib was the first that gained FDA approval in February 2015, intended for use in men and postmenopausal women [18]. In March 2017, ribociclib was approved for women regardless of menopausal status [19]. Lastly, abemaciclib gained approval in February 2018 for postmenopausal women [19].

### 3.1. Palbociclib

Palbociclib is a small molecule inhibitor of CDK4 and CDK6 that is taken orally [20]. A phase I trial (NCT00141297) tested for the optimal dosage amounts and schedule. The first group of patients with various tumor types received between 100 and 225 mg palbociclib daily for two weeks at a time, followed by one week off, as suggested due to preclinical data. A total of 32% of patients in this trial had a positive response, and the higher doses were noted to be more effective [21]. The other group received between 25 and 150 mg palbociclib in a three week on/one week off schedule. In this case, 27% of patients responded and the most benefit was seen with the highest dose [22]. Higher doses of palbociclib, however, also led to more significant adverse events, including neutropenia, leukopenia, anemia, fatigue, thrombocytopenia, arthralgia, and nausea [20,23]. In addition, various cutaneous side effects have been reported [24]. To best cope with this, the 21 day on/7 day off schedule of 125 mg daily was recommended for future studies [25].

In the PALOMA-1 phase II clinical trial (NCT00721409) with hormone receptor-positive (HR+)/HER2- breast cancer patients, progression-free survival (PFS) after treatment with either 2.5 mg letrozole daily or 2.5 mg letrozole daily and 125 mg palbociclib in 3 week on/1 week off cycles was monitored. These patients were allowed to have previous treatment with an aromatase inhibitor. Patients treated with the combination therapy had a median PFS of 26.1 months, as opposed to 5.7 months for the letrozole-only group [20]. This study provided strong evidence that this CDK4/6 inhibitor significantly aids in prolonging remission in patients with ER+ breast cancer.

The PALOMA-2 trial (NCT01740427) was a phase III study that evaluated the efficacy of 125 mg palbociclib and 2.5 mg letrozole compared to 2.5 mg letrozole alone in HR+/HER2− patients who had not received prior treatment. The group treated with the combination including palbociclib has a median PFS of 24.8 months, while the letrozole-only group’s PFS was only 14.5 months [26]. Altogether, these two clinical trials allowed for palbociclib to be approved for use in postmenopausal women with advanced breast cancer, whether or not they had received treatment in the past with an aromatase inhibitor.

The PALOMA-3 clinical trial (NCT01942135) was a phase III trial that examined the effects of combining 125 mg palbociclib with 500 mg fulvestrant, in advanced HR+/HER2− breast cancer. This study found that PFS increases from 4.6 months to 9.5 months when fulvestrant is paired with palbociclib instead of a placebo [23]. Importantly, this regimen was able to overcome resistance in both premenopausal and postmenopausal women who previously were only administered antiestrogens [27]. Overall, the PALOMA-3 trial led to the approval of palbociclib as a second-line treatment option for advanced ER+ breast cancer patients, regardless of menopausal status or prior antiestrogen treatment [19].

The PALINA trial (NCT02692755) investigated the use of palbociclib in African American women with HR+/HER2− advanced breast cancer. Looking into this subset of patients is important because they are underrepresented in the PALOMA trials. Additionally, many have a genetic polymorphism in the Duffy antigen receptor for chemokines that increases their prevalence of benign ethnic neutropenia [28]. Patients with or without the variant were given palbociclib and endocrine therapy had their response tracked over 12 months. Those with the Duffy null variant experienced more instances of severe neutropenia (72.2%) compared to wild-type (WT) patients (23.1%) [28]. Consequently, a dose reduction was necessary in 55.6% of the Duffy null women, as opposed to 7.7% of WT women. Furthermore, they also had a lower clinical benefit rate of 66.7% compared to 84.6% in WT patients. This study showed that palbociclib is effective and tolerable in African American women, but showed that patients with Duffy null status need to be carefully monitored for severe neutropenia and have their palbociclib dose adjusted as necessary.

Continuous administration of palbociclib has not been investigated due to the development of severe hematological adverse effects that arise. Fortunately, the 1-week interruption allows enough time for the bone marrow to recover. While initially seen as a disadvantage to this drug, the 7-day rest actually allows palbociclib to be synergized with other drugs that can further disrupt the cell cycle [4]. At the start of the break from palbociclib treatment, cells are synchronized in their cell cycle progression. Chemotherapeutic agents that damage the DNA are likely to be highly effective at this point because the cells are at a susceptible time in the cell cycle [4]. This concept is currently being tested in the clinic.

Palbociclib has a limited ability to penetrate the blood–brain barrier, since this drug can be used as a substrate by breast cancer resistance protein (BCRP) and P-glycoprotein (P-gP) [25]. This has caused some skepticism in how well it can target metastases. Nevertheless, the potential of palbociclib in treating breast cancer patients with brain metastases is being evaluated in a clinical trial (NCT02774681). It is important to note that due to differences in how palbociclib is metabolized due to genetic variants of Cytochrome P450 Family 3 Subfamily A Member 4 (CYP3A4) and Sulfotransferase Family 2A Member 1 (SULT2A1) in certain patients, Asian patients in particular are more likely to experience serious neutropenia [25]. As a result of this and the PALINA trial, patients need to have their dosages carefully considered and adjusted as necessary based on any adverse events.

### 3.2. Ribociclib

Ribociclib is an oral, small molecule inhibitor of CDK4/6 that dephosphorylates Rb [14]. As a result, the E2F transcription factors are not able to induce transcription, and the cell arrests in the G1 phase. In a phase I clinical trial (NCT01237236) including patients with multiple cancer types, 20 ER+ breast cancer patients had tolerance of 600 mg per day of ribociclib tested. Adverse effects included myelosuppression, grade 3/4 neutropenia, and thrombocytopenia [14]. Less serious effects include fatigue, nausea, vomiting, and diarrhea. To increase tolerance, it was recommended that a 1-week break should be given for each 3-week continuous period of taking the drug, as is performed with palbociclib.

The MONALEESA-2 phase III trial (NCT01958021) treated HR+/HER2− postmenopausal patients with either 600 mg ribociclib daily and 2.5 mg letrozole or letrozole alone. After follow-up, it was determined that the average PFS in ribociclib-treated patients was 25.3 [29]. The letrozole-only group had a median PFS of 16.0 months. As a result, ribociclib was suggested for use in advanced postmenopausal patients.

The MONALEESA-3 trial (NCT02422615) continued looking into the efficacy of ribociclib. Men and postmenopausal women with advanced HR+/HER2− breast cancer were split into two groups: 2/3 of patients received 600 mg ribociclib and 500 mg fulvestrant, while 1/3 received fulvestrant alone. Importantly, this trial allowed both patients who received prior endocrine therapy and had metastases. Patients remained on either treatment for one year. The overall survival (OS) for the group treated with ribociclib/fulvestrant was 51.8 months, compared to 39.7 months for the fulvestrant-only group [30]. These data suggested that ribociclib and fulvestrant could help to extend survival in both men and women with HR+ breast cancer.

The first trial to test the efficacy of ribociclib in premenopausal women was the phase III MONALEESA-7 trial (NCT02278120). HR+/HER2− patients regardless of previous treatment with chemotherapy or endocrine therapy were allowed. Patients were given 600 mg ribociclib or a placebo in combination with 20 mg tamoxifen, 2.5 mg letrozole, or 1 mg anastrozole. Furthermore, all patients were given 3.6 mg goserelin injections monthly, which is needed in premenopausal women to halt estrogen production in the ovaries [31]. The average PFS in the ribociclib group was 23.8 months, compared to 13.0 months in the control group [32]. This study supported the approval of ribociclib for premenopausal patients.

Based on the result of the aforementioned studies, ribociclib is given to patients in a 3 week on, 1 week off schedule. The 7-day break is necessary because continuous administration of ribociclib causes severe hematological toxicities [25]. The one-week break helps to minimize these effects and allows for marrow recovery while still experiencing tumor growth suppression [4]. As is the case with palbociclib, this break provides an opportunity for other drugs, such as DNA-damaging chemotherapies, to synergize well due to cells re-entering at a highly susceptible point in the cell cycle [4]. Additionally, preclinical studies have shown that ribociclib has a limited ability to penetrate the blood–brain barrier, since it is also a substrate for BCRP and P-gP [25]. However, the potential use of ribociclib in treating brain metastases is currently being investigated in a clinical trial (NCT02933736).

### 3.3. Abemaciclib

Abemaciclib is a small-molecular inhibitor of CDK4 and CDK6, but it also has strong activity against Cyclin-dependent Kinase 9 (CDK9). In contrast to palbociclib and ribociclib, it has about five times more potent activity against CDK4 [25]. A phase I trial (NCT02308020) was used to evaluate the maximum tolerated dose of abemaciclib in patients with various solid tumor types. Patients were split into a once daily group that received between 50 and 225 mg, while the twice daily group received between 75 and 275 mg [12]. In this study, adverse effects for abemaciclib included severe fatigue, diarrhea, vomiting, leukopenia, neutropenia, thrombocytopenia, anemia, and increased creatinine levels. These effects are dose-dependent and reversible. The maximum dose that was well-tolerated by patients was 200 mg every 12 h. Based on the data achieved in this trial, future trials utilized the twice per day schedule with doses between 150 and 200 mg.

Abemaciclib was first approved as a second-line treatment if combined with fulvestrant based on the phase III MONARCH-2 study (NCT02107703). HR+/HER2− patients who progressed while taking endocrine therapy were given either 150 mg abemaciclib twice daily with fulvestrant or fulvestrant alone. Treatment was administered continuously over the course of 15 months. At the end of the treatment period, the group who received abemaciclib had a PFS of 16.4 months, while the fulvestrant only group had a median PFS of 9.3 months [33]. The experimental group had an objective response rate of 48.1% compared to 21.3% for the control group. Considering its efficacy, 150 mg abemaciclib was deemed to be tolerable and approved as a treatment option for when progression on endocrine therapy occurs.

Abemaciclib was also approved for use with AIs based on the MONARCH-3 phase III trial (NCT02246621). This study focused on postmenopausal HR+/HER2− patients previously treated with endocrine therapy. Response to 150 mg abemaciclib or placebo twice per day combined with either anastrozole or letrozole was noted. The median PFS of the placebo group was 14.7 months, while the median PFS of the abemaciclib group was not even reached [34]. The efficacy of abemaciclib is further highlighted by the 59% response rate, compared to 44% in the placebo-treated group. Overall, abemaciclib in combination with an AI was deemed effective and was given approval for use in women who progressed on first-line endocrine therapy.

The phase II MONARCH-1 trial (NCT02102490) was the first clinical trial to test abemaciclib as a single agent. Patients with HR+/HER2− metastatic breast cancer who had progressed while on or after completing endocrine treatment and chemotherapy were given either 200 mg abemaciclib or a placebo twice per day. After 12 months of treatment, the objective response rate was 19.7% for those receiving abemaciclib [35]. Additionally, the average PFS was 6 months, and the median OS was 17.7 months. Considering the severity of disease in this patient cohort, abemaciclib was considered to be highly effective.

Interestingly, abemaciclib is the one CDK4/6 inhibitor that has been relatively successful in HER2+ patients, which was first suggested based on preclinical studies [12]. Women enrolled in the monarcHER trial (NCT02675231) had HR+/HER2+ advanced or recurrent breast cancer that did not respond to two or more prior HER2-targeted therapies. This trial tested the efficacy of abemaciclib by dividing patients into three groups. Group 1 received 150 mg abemaciclib twice daily, 8 mg/kg trastuzumab (an anti-HER2 monoclonal antibody) on day 1 followed by 6 mg/kg monthly, and 500 mg fulvestrant 3 times in the first month followed by once every subsequent month. Group 2 was treated with the same schedule, but only received abemaciclib and trastuzumab, while group 3 was given trastuzumab and their physician’s choice of a standard of care chemotherapy regimen. The PFS of group 1 was 8.3 months, compared to 5.7 months for both groups 2 and 3 [36]. Overall, this study showed that the abemaciclib/fulvestrant/trastuzumab combination is effective, even in patients with advanced HER2+ disease.

Abemaciclib is the only CDK4/6 inhibitor that can be given on a continuous schedule with no break. Furthermore, it is given twice per day as opposed to once per day. This is due to the fact that studies have shown abemaciclib to reach a saturation of absorption; by breaking the recommended doses in half and taking it twice per day, the absorption is improved [25]. Luckily, more than one benefit is achieved from this specific dosing schedule. Giving patients abemaciclib continuously without a week-long break is more efficient at reducing tumor volume than it is with the break [37]. Fascinatingly, these studies exemplify that this drug is the only CDK4/6 inhibitor to be well tolerated when taken daily with no off-time. Since diarrhea is an incredibly common side effect, most patients also take an antidiarrheal with abemaciclib [35]. The high lipophilicity of abemaciclib allows it to be the only CDK4/6 inhibitor that can easily penetrate both the blood–brain barrier and breast tissue to decrease brain tumor growth [25]. Thus, abemaciclib is theoretically the best equipped CDK4/6 inhibitor for use in breast cancer patients with brain metastases, which is further being investigated in an ongoing trial (NCT02308020).

These three CDK4/6 inhibitors are considered to have equal efficacy, though they have never been directly compared. Each can be prescribed as a first-line or second-line treatment in combination with endocrine therapy for use in patients with HR+/HER2− breast cancer at an advanced stage [19]. However, the optimal positioning of CDK4/6 inhibitors has not yet been concluded. A phase III clinical trial, the SONIA study (NCT03425838), is investigating the best use of CDK4/6 inhibitors in patients with HR+/HER2- advanced breast cancer [38]. Patients in this study will be split into two groups. The first group will receive a CDK4/6 inhibitor along with a non-steroidal aromatase inhibitor as a first-line treatment. When progression eventually occurs, patients will be administered fulvestrant. The second group will initially receive a non-steroidal aromatase inhibitor, but when progression occurs they will receive a CDK4/6 inhibitor in combination with fulvestrant. At the end of the trial, which will conclude when there are sufficient data about secondary progression points in each arm, patient outcomes will be evaluated to determine if CDK4/6 inhibitors are more effective when included in a primary or secondary treatment regimen.

## 4. Emergence of Intrinsic and Acquired Resistance to CDK4/6 Inhibitors

Analysis of circulating tumor DNA of patients in the PALOMA-3 trial displayed that there are two distinct types of resistance that can arise due to combination treatment of antiestrogens and CDK4/6 inhibitors. Interestingly, mutations that allowed for resistance depended on the treatment administered and occurred at specific times during treatment. Different genetic mutations in circulating tumor cells were seen between the group receiving fulvestrant and palbociclib and the group just receiving fulvestrant, revealing that different mechanisms are needed to grow in the presence of antiestrogens versus what is needed to bypass CDK4/6 inhibitors. However, mutations also differed within each group depending on if the cancer progressed early or late after treatment [39]. This suggests that the mutations that cause initial resistance are different than those that are needed for resistance that occurs after a period of successful treatment.

### 4.1. Intrinsic

Resistance that occurs shortly after treatment begins and causes the patient to have no response is referred to as intrinsic resistance. About 20% of breast cancer patients treated with CDK4/6 inhibitors never respond to treatment [40]. These patients’ tumors already have mutations present that allow them to circumvent that action of the CDK4/6 inhibitor and proliferate in the presence of the drug. Many mechanisms of intrinsic resistance have been identified to date, and they all seem to involve the activation of the cyclin D–CDK4/6–Rb pathway [41]. Intriguingly, intrinsic resistance to CDK4/6 inhibition is present in all tumors that have mutated Rb [42]. The mechanism by which this type of resistance occurs will be explained in detail below.

### 4.2. Acquired

Patients who initially respond but progress late on treatment are categorized as having acquired resistance. Driver mutations that allow for this are the result of clonal evolution [39]. Note that many different mechanisms can lead to acquired resistance to CDK4/6 inhibitors, including cyclin D–CDK4/6–Rb activation, the activation of other proliferation pathways, the alteration of the tumor microenvironment, and the adjustment of the tumor metabolism [41].

In vitro experiments treating MCF7 and T47D cells with palbociclib display the ease with which breast cancer cells can become resistant to CDK4/6 inhibition. After 24 h of treatment, cell cycle arrest occurs. After 72 h, cells begin to enter a senescent state and undergo apoptosis, displaying that the cells are initially responding to the treatment. However, if treatment extends past 72 h, some cells are able to re-enter the cell cycle and continue growth [43]. After more time passes, these cells would be the ones that compose the tumor in a patient, since they are the ones that can survive in the presence of the CDK4/6 inhibitor. While this experiment exemplifies resistance that develops quickly, resistance in humans takes a bit longer. Regardless, the experiment accurately depicts how cells must adapt in order to survive in the presence of drugs they are not inherently resistant to.

There is ample evidence from the clinic that acquired resistance is all too common. Within 2 years of beginning treatment in the PALOMA-2 trial, over 30% of enrolled patients developed resistance to palbociclib [26]. After 40 months, more than 70% of patients in the palbociclib and letrozole arm of the trial had progressed on treatment. As time from the initiation of treatment goes on, more and more patients will develop resistance; eventually, acquired resistance develops in all patients treated with CDK4/6 inhibitors [41]. Consequently, discovering the mechanisms behind this resistance is important because it will better enable clinicians to target it with novel treatments.

## 5. Mechanisms of Resistance to CDK4/6 Inhibitors

**Figure 1:**
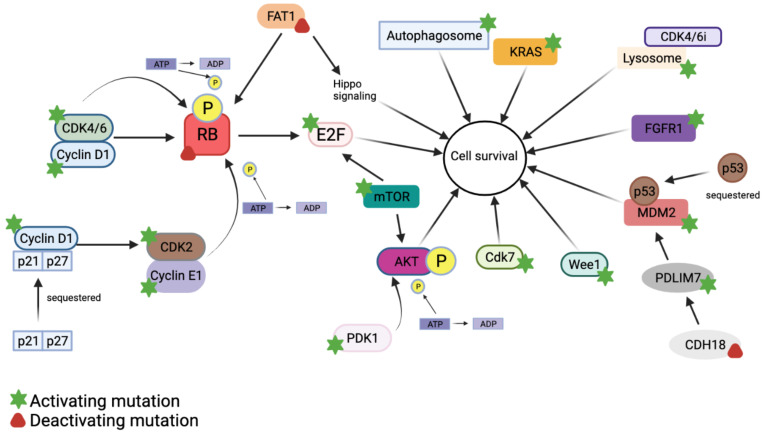
Activating and deactivating mutations identified to date that allow cells to survive in the presence of CDK4/6 inhibitors. Activation of cyclin D1, CDK4/6, CDK2, cyclin E1, E2F, PDK1, mTOR, CDK7, Wee1, PDLIM7, MDM2, FGFR1, KRAS, the lysosome, and the autophagosome can help cells push through the cell cycle when CDK4/6 are pharmacologically inhibited. Deactivation of RB, FAT1, and CDH18 can lead to similar effects on cell cycle progression and overall cell survival. Mutations in each of these proteins and cellular components help the cell to survive via a variety of mechanisms, which are detailed in Section 5.

Many mechanisms of resistance to CDK4/6 inhibitors have been demonstrated in both preclinical and clinical studies. These are summarized in Figure 1.

### 5.1. Cyclin D1–CDK4/6–Rb Activation

Upregulation of the cyclin D1–CDK4/6–Rb pathway is arguably the most straightforward cause of CDK4/6 inhibitor resistance. The main way that this pathway is upregulated is via direct overexpression of CDK4 or CDK6. Yang et al. [44] sequenced cells resistant to abemaciclib and found that many contained a CDK6 amplification. Other than amplification, an activating mutation in CDK4 or CDK6 can cause the pathway to be overactive.

Loss of Rb can also cause this pathway to be upregulated. Using an ex vivo model to study palbociclib response in different human breast cancers, Dean et al. [45] determined that intrinsic resistance to this inhibitor is present when Rb has a loss-of-function mutation. The inactivation of Rb confers resistance because Rb can no longer prevent transition from G1 to S, so inhibiting CDK4/6 has no effect on the cell [45]. Moreover, data from the PALOMA-3 trial showed that deactivating mutations in RB1 that allow for resistance only occur when patients are treated with combination antiestrogen and CDK4/6 inhibitor treatment. Interestingly, no Rb mutations were observed in patients treated only with fulvestrant [39]. Guarducci et al. [46] echo the importance of WT Rb for maintaining sensitivity to CDK4/6 inhibition. Their gene expression profiles of seven ER+ cell lines found that Rb is reduced in cells that acquire resistance to palbociclib, though Rb only completely lost expression in one of the seven lines. Fortunately, fully inactivating mutations of Rb in ER+ breast cancer patients are relatively rare. One study that looked at genetic profiles of 127 patients with invasive lobular carcinoma found that only 3.9% of these tumors contained entirely nonfunctional Rb [47]. This finding is important because functional Rb is necessary for patients to have any initial response to a CDK4/6 inhibitor.

The activity of the cyclin D1–CDK4/6–Rb pathway can also be altered when there is upregulated or overactive E2F. The presence of too much E2F can overpower Rb, making Rb unable to bind all of the E2F in the cell. Thus, E2F will be more transcriptionally active. By analyzing gene signatures in The Cancer Genome Atlas, it was identified that increased E2F Transcription Factor 1 (E2F1) and increased E2F Transcription Factor 2 (E2F2), two members of the E2F family, were correlated with a loss of function of Rb in ER+ breast cancers [48]. Furthermore, these same cell lines that had a loss of Rb were also much more likely to be resistant to palbociclib. Rb signature was also a prognostic factor, as patients who lost Rb function had a significantly poorer OS. Altogether, these data suggest that E2F expression can inhibit a therapeutic response to CDK4/6 inhibition in ER+ breast cancers.

### 5.2. Cyclin E1/CDK2 Activation

CCNE1 is the gene that codes for cyclin E1. It is downstream of the cyclin D1–CDK4/6–Rb pathway. Cyclin E can bind to Cyclin-dependent Kinase 2 (CDK2), a G1/S phase regulator, to phosphorylate Rb independently of CDK4/6 and cyclin D1 [49]. Therefore, the activation of cyclin E1 or CDK2 allows cells to bypass the action of CDK4/6 inhibitors to encourage growth and proliferation.

Using seven different ER+ breast cancer cell lines, researchers measured the differential expression of genes and proteins before and throughout the course developing resistance to palbociclib [46]. After becoming resistant over the course of 10–27 weeks, gene expression analysis identified that all cell lines overexpressed cyclin E1 due to amplification or upregulation. Furthermore, the testing of 38 different breast cancer cell lines revealed that cells that are not responsive to palbociclib have high cyclin E expression [50].

Herrera-Abreu et al. [43] compared copy number alterations of CCNE1 in MCF7, T47D, and corresponding palbociclib-resistant cell lines using a BrdUrd incorporation-ELISA assay. Interestingly, CCNE1 is amplified in the cells that have acquired resistance to palbociclib. These resistant cells regained sensitivity, as measured by cell cycle arrest and a reduction in growth, to palbociclib when they co-treated with siRNA targeting cyclin E1 or CDK2. This observation suggests that cyclin E1 is upregulated via CDK2 activation in the resistant cells, since CDK2 can bind and induce proliferation when CDK4/6 are repressed. 

Patient data also support that cyclin E1 can be implicated in resistance to CDK4/6 inhibition. The NeoPalAna trial (NCT01723774) evaluated the efficacy of neoadjuvant palbociclib and anastrozole versus monotherapy with anastrozole in patients with ER+ breast cancer [51]. Using Agilent microarray to view the differential gene expression of tumor samples before and after treatment, it was determined that the expression of CCNE1 was significantly increased in patients treated with palbociclib who become resistant, while this change was not observed in patients treated with anastrozole alone.

### 5.3. PI3K–AKT Activation

Many studies have demonstrated the effect that overactivity of the PI3K–AKT pathway can have on how tumors respond to CDK4/6 inhibitors. Though not specifically analyzing ER+ breast cancer, one study using patient-derived xenografts (PDX) of melanoma to analyze resistance to CDK4/6 inhibitors determined that the PI3K–AKT pathway was activated in the intrinsically resistant models [52]. CDK4/6 inhibitors cause the cells to upregulate cyclin D1, which binds to p21 and p27, two downstream regulators of the PI3K–AKT pathway. When these two CDK inhibitors are sequestered, they cannot bind to CDK2. As a result, CDK2 is active and allows for proliferation. This mechanism was verified by showing that cells have an improved response to CDK4/6 inhibition when p21 is restored.

3-phosphoinositide dependent kinase 1 (PDK1) works in the PI3K–AKT pathway to fully activate AKT Serine/Threonine Kinase (AKT) via phosphorylation [53]. This induces a signaling cascade that promotes growth and survival [54]. A high throughput in vitro siRNA screen in breast cancer cells identified that PDK1 knockdown increases the sensitivity of cells to CDK4/6 inhibition [53]. Additionally, in vitro use of a PDK1 inhibitor in combination with ribociclib helps resistant cells to respond. As a result, PDK1 is implicated in the resistance of ER+ breast cancer cells to CDK4/6 inhibitors.

Activating mutations in two AKT isoforms, AKT Serine/Threonine Kinase 1 (AKT1) and AKT Serine/Threonine Kinase 3 (AKT3), were also identified to contribute to resistance in this siRNA screen. It showed that when these two kinases are knocked down, sensitivity to CDK4/6 inhibitors is restored [53]. Furthermore, pathway analysis comparing MCF7 and T47D sensitive and resistant cells identified that increased activity of AKT1 is present in the cells resistant to palbociclib [55]. Additionally, combining palbociclib with an AKT inhibitor helped to overcome resistance in vitro, confirming that AKT activity helps cells confer resistance to CDK4/6 inhibition.

### 5.4. KRAS Activation

Kirsten Rat Sarcoma Viral Oncogene Homolog (KRAS), the most frequently mutated RAS isoform, has roles in processes such as apoptosis, growth, and differentiation [56]. Overactive K-Ras can result in the propagation of aberrant growth signals and has been noted to lead to drug resistance in many types of cancer [57]. This phenomenon was first seen in breast cancer in a study that analyzed blood samples from 106 ER+ metastatic breast cancer patients who received fulvestrant in combination with palbociclib. Notably, these patients all had prior sensitivity to endocrine therapy. Patients who developed KRAS mutations, determined via liquid biopsy, became resistant to the treatment. In fact, their average PFS during treatment was 3 months, while the PFS of WT KRAS patients had not even been reached by the 18-month follow-up [57]. Patients with increased numbers of circulating cells containing KRAS mutations also had more metastatic sites. Most patients with KRAS-mutant cancers also have high cyclin D1 expression [58]. This further helps to explain how KRAS can cause abnormal growth signaling in the presence of CDK4/6 inhibitors. To date, no successful therapies have been found to target KRAS mutations in ER+ CDK4/6 inhibitor-resistant breast cancer.

### 5.5. Autophagy and Lysosomal Activity

Autophagy is used in cells when they are undergoing stress. It is a lysosome-mediated process that recycles cellular components into an energy source. When resistant cells are treated with the drug they do not respond to, autophagic flux increases [59]. Autophagy has previously been shown to be upregulated in resistant cells in triple-negative breast cancer.

When ER+ breast cancer cells are treated with palbociclib, they increase their reliance on autophagy [60]. Transcriptomic profiling of MCF7 cells that had become resistant to palbociclib compared to sensitive parental cells showed an upregulation of many genes involved in autophagy [61]. The resistant cells also formed more autophagosomes, further highlighting that ER+ breast cancer cells upregulate autophagy to cope with pharmacological CDK4/6 inhibition. Autophagy also helps cells to cope with CDK4/6 inhibition by blocking apoptosis [61]. By preventing apoptosis, cells that should be killed as a result of CDK4/6 inhibition are able to remain alive.

One study investigated the role of lysosomal activity in triple-negative breast cancer cells, which are resistant to CDK4/6 inhibitors. Fascinatingly, there is a subset of triple-negative breast cancer cells that actually does rely on CDK4/6 for proliferation, which implies that targeting CDK4/6 could be a therapeutic option for such patients. When questioning why these cells do not respond to CDK4/6 inhibitors, it was determined that cells upregulate lysosomes, which help to sequester the CDK4/6 inhibitors [62]. This was confirmed by coadministration of CDK4/6 inhibitors with lysosomotropic or lysosome destabilizers, which helps to sensitize the resistant cells. Additionally, administering an altered CDK4/6 inhibitor that evades the lysosome also increases sensitivity. Though this work has not been repeated in ER+ breast cancer cells, it is possible that resistance to CDK4/6 inhibitors in ER+ breast cancer is the result of increased lysosomal segregation. Further, targeting lysosomes can potentially be a mechanism of bypassing this resistance. To confirm this possibility, these experiments must be repeated in models of ER+ breast cancer.

### 5.6. FAT1 Loss

FAT Atypical Cadherin 1 (FAT1) is a tumor suppressor that is involved in development, proliferation, migration, and invasion [63]. Its dysregulation has been implicated in many cancers. To identify genes that could be responsible for resistance to CDK4/6 inhibitors, Li et al. [64] performed a sequencing screen of cancer-associated genes on 348 patient-derived ER+/HER2− breast cancer samples. These patients were then treated with CDK4/6 inhibitors, and their response was compared to the original genetic profile of their tumors. Interestingly, patients with FAT1 alterations had a shorter PFS (2.4 months) than patients with this gene unmodified (10.1 months). Subsequent in vivo experiments confirmed this role of FAT1 loss. MCF7-implanted xenografts with a FAT1 knockdown or knockout experienced much less sensitivity to 50 nM abemaciclib than mice with WT FAT1. Western blot analysis on these samples showed that decreased FAT1 allows for some phosphorylation of Rb and induced CDK6 expression, whereas the FAT1 WT cells experience a completely blocked Rb phosphorylation and less CDK6 expression [64]. qPCR also revealed that FAT1 loss results in increased Hippo signaling. Moreover, Yes1 Associated Transcriptional Regulator (YAP1) and Transcriptional Coactivator with PDZ-binding motif (TAZ) knockdown suppress CDK6 signaling and restore CDK4/6 inhibitor sensitivity in these cells. Therefore, it was determined that FAT1 loss increases Hippo signaling and induces strong CDK6 activity, rendering these cells insensitive to treatment.

### 5.7. FGFR1 Activation

Fibroblast growth factor receptor 1 (FGFR1) is a tyrosine kinase family protein. Its signaling is vital for cellular processes such as migration, proliferation, differentiation, and survival [65]. FGFR1 is amplified or overexpressed in 15% of ER+ breast cancers [66]. Not surprisingly, FGFR1 mutations have already been implicated in the resistance of ER+ breast tumors to endocrine therapy. To investigate its possible role in resistance to CDK4/6 inhibitors, FGFR1 was one of many kinases that were overexpressed in MCF7 and T47D cells treated with fulvestrant and ribociclib. Formisano et al. [66] found that overexpressing FGFR1 leads the cells to be less responsive to this treatment. To verify this suspicion, the same resistant cells overexpressing FGFR1 were given lucitanib, an FGFR inhibitor. Indeed, this treatment reversed the previously observed resistance. The same results were achieved in xenograft models using another FGFR1 inhibitor, erdafitinib. This study also evaluated the FGFR1 status of patients from the MONALEESA-2 trial, and found that patients with a FGFR1 amplification experienced a shorter PFS than patients with WT FGFR1 [66]. Hence, FGFR1 amplification or upregulation has since been implicated in CDK4/6 inhibitor resistance.

### 5.8. MDM2 Dysregulation

Though most breast cancers do not have mutated p53, it can often be dysregulated through the activation of regulatory proteins such a Mouse Double Minute 2 Homolog (MDM2) [67]. MDM2 interacts with p53 to inhibit its action through sequestration and signaling for its degradation. Therefore, higher MDM2 activity can prevent DNA repair, encourage inappropriate cell cycle progression, and stop the apoptosis of severely damaged cells [68]. Intriguingly, ER+ breast cancers are more likely to have higher levels of MDM2. Cells resistant to CDK4/6 inhibitors are unable to properly induce senescence, and the interruption of the p53 pathway due to upregulated MDM2 can cause this resistance to occur [67,69]. MDM2 is regulated by PDZ and LIM Domain 7 (PDLIM7), which helps to stabilize MDM2. When more cadherin 18 (CDH18), a type II cadherin, is present, PDLIM7 is sequestered and cannot stabilize MDM2 [70]. In accordance with this mechanism, CDH18-negative patients have a worse PFS (9 weeks) compared to CDH18-positive patients (17.9 weeks) when treated with palbociclib in clinical trials [70]. Furthermore, the OS of CDH18-negative patients was 19.8 months versus 42.4 months for CDH18-positive patients. In short, CDH18-negative patients have higher MDM2 activity, which can allow them to circumvent CDK4/6 inhibition. Targeting this pathway by inhibiting downstream MDM2 is thus a feasible way to reverse resistance to CDK4/6 inhibition.

### 5.9. mTOR Activation

In ER+ breast cancer cells that are resistant to CDK4/6 inhibitors, the mammalian target of rapamycin (mTOR) pathway is commonly mutated [71]. The mTOR kinase forms two separate complexes, mTORC1 and mTORC2. mTOR is involved in many processes that can influence cell growth, such as control of cell cycle, size, proliferation via mitogen and nutrient signaling, and autophagy. Further complicating the functions of mTOR is that it is partially regulated by the PI3K–AKT pathway. Moreover, Michaloglou et al. [71] used qPCR to determine that vistusertib, an mTORC1/2 inhibitor, leads to a decrease in E2F-mediated transcription. Thus, mTOR activity is responsible for upregulating this. As already explained, cells resistant to CDK4/6 inhibitors have been shown to increase reliance on E2F. This suggests that cells that upregulate mTOR leverage an increase in E2F-mediated transcription to resist the action of CDK4/6 inhibitors, and targeting mTOR can reverse E2F-dependent cell proliferation. 

### 5.10. Increased G2M Activity: WEE1 and CDK7 Overexpression

A defective G1 checkpoint is caused by therapy with CDK4/6 inhibitors. To cope with the loss of this checkpoint, cells rely on the G2 checkpoint to fix any DNA damage that may be present prior to division [72]. Through an analysis of ER+ primary breast tumors from The Cancer Genome Atlas database, it was determined that an increased G2M pathway score correlates with a higher expression of genes associated with proliferation, such as Myc, mitotic spindle, and E2F. The G2M score is a measure of activity of the G2/M cell cycle checkpoint. As previously stated, cells treated with CDK4/6 inhibitors rely on the G2/M checkpoint for correction. Increased G2/M activity in highly proliferative cells further helps to explain how cells with suppressed G1/S activity can continue to grow [73].

**Figure 2:**
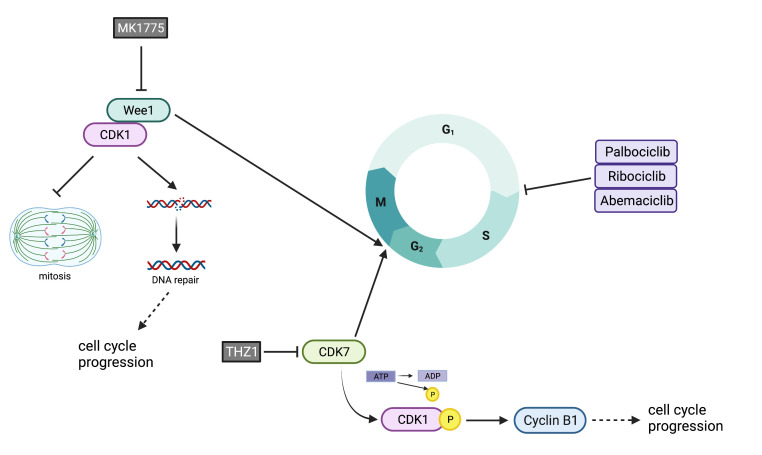
Increasing reliance on the G2/M checkpoint allows cells to bypass the G1/M deficiencies caused by CDK4/6 inhibitors. Wee1 works at the G2/M checkpoint to stall mitosis and induce DNA repair so that cells can progress in the presence of CDK inhibitors. MK1775 works here to inhibit the action of Wee1, thus preventing repair from occurring and so the cell cannot continue through division. Additionally, at G2/M, CDK7 phosphorylates CDK1 to cause cell cycle progression via cyclin B1. THZ1 is an agent that inhibits CDK7 to prevent this method of cell cycle progression.

Kinase rewiring is required for cells to develop resistance to CDK4/6 inhibitors. CDK4/6 inhibitor-resistant cells that develop from sensitive parental cells have an elevation of cyclins and CDKs that are active at the G2/M phase [74]. The WEE1 G2 Checkpoint Kinase (WEE1) and Cyclin-dependent Kinase 7 (CDK7), both of which regulate G2/M, have been connected to resistance in palbociclib-resistant cells (Figure 2).

Wee1, in coordination with Cyclin-dependent Kinase 1 (CDK1), is one cell cycle regulator that acts at the G2/M checkpoint to prevent damaged cells from progressing through division. This tyrosine kinase regulates the timing of mitosis so that damaged cells have the opportunity to repair damage prior to dividing [72]. An siRNA kinome knockdown in MCF7 and T47D cells resistant to palbociclib showed that Wee1 knockdown helps to reverse resistance [75]. The role of Wee1 was further validated using MK1775, a Wee1 inhibitor. This finding helps to further implicate Wee1 overexpression as a resistance mechanism to CDK4/6 inhibitors and confirm that resistant cells rely more heavily on the G2/M checkpoint than sensitive cells do.

CDK7, which has multiple roles throughout the cell cycle, is involved in G2/M cell cycle progression. Here, it is needed to phosphorylate CDK1 and increase the expression of cyclin B1 [76]. These components are necessary for G2/M progression. The siRNA kinome knockdown by Martin et al. [75] exemplifies the importance of CDK7 in palbociclib-resistant cells. Resistant cells that have CDK7 knocked down develop an increased sensitivity to palbociclib. THZ1, a CDK7 inhibitor, was used to validate that CDK7 downregulation can help overcome resistance. Altogether, these data support that resistant cells increase reliance on CDK7 to bypass G1/S inhibition.

## 6. Possible Therapies to Target Intrinsic or Acquired Resistance to CDK4/6 Inhibitors

Though not all of the above mechanisms have been successfully targeted, multiple drugs that reverse resistance in CDK4/6-resistant tumors have been identified to date.

### 6.1. Antiestrogens

When CDK4/6 inhibitors began to be investigated in the clinic, the standard of care was antiestrogen therapy with either a SERM, a SERD, or an AI. So, when clinical trials began to test CDK4/6 inhibitors, they were used in combination with this standard of care. However, initial trials did not investigate the efficacy of CDK4/6 inhibitors alone; thus, the efficacy of antiestrogens in this context was relatively unknown. The phase II TREnd trial (NCT02549430) included postmenopausal women with HR+/HER2− metastatic breast cancer who had previously progressed on endocrine therapy. They were either given 125 mg palbociclib daily for 3 weeks per 28-day cycle or the same regimen of palbociclib but with the continued endocrine therapy they were previously receiving [77]. The median PFS of patients on palbociclib alone was 6.5 months, while patients on combination therapy experienced a longer benefit of 10.8 months. Notably, this advantage was only seen in patients who had received prior endocrine therapy for more than 6 months.

Another trial using abemaciclib sought to see if this difference was observed using a different CDK4/6 inhibitor, which is important because abemaciclib is the only one currently approved in some cases as a monotherapy. The nextMONARCH trial included HR+/HER2- metastatic breast cancer patients who previously received chemotherapy and divided them into three groups [78]. The monotherapy group received 150 mg abemaciclib every 12 h. Another group was given 200 mg abemaciclib every 12 h, along with loperamide to help manage the common adverse effect of early-onset diarrhea that is experienced with high doses of abemaciclib. The third group received 150 mg abemaciclib every 12 h and 20 mg tamoxifen. Interestingly, loperamide did not seem to provide any significant benefit concerning the incidence and severity of diarrhea. There was a minimal, but statistically nonsignificant, PFS benefit of 9.1 months in the group receiving abemaciclib and tamoxifen compared to 7.4 months in the group receiving 200 mg of abemaciclib. The PFS of the group receiving 150 mg abemaciclib was slightly less at 6.5 months. It is vital to mention that this trial did not look at patients who were resistant to CDK4/6 inhibitors. Therefore, the minimal benefit seen in this trial might be amplified when resistant patients are given endocrine therapy concurrently with abemaciclib. To address this possibility, future trials need to focus on this drug combination in this subset of patients.

No trials or preclinical studies have yet officially investigated if adding hormonal therapy to ribociclib treatment helps to prolong the development of resistance to ribociclib. Though ribociclib has been shown to be quite similar to palbociclib, studies to investigate this important question are still warranted and will hopefully be initiated in the near future.

### 6.2. PI3K Inhibitors

PI3K signaling is involved in growth, metabolism, proliferation, and survival, among other functions within the cell. Interestingly, acquired resistance to CDK4/6 inhibition is correlated with higher levels of PI3K signaling. O’Brien at al. [79] used a reverse phase protein array in both in vitro and in vivo models of CDK4/6-resistant ER+ breast cancer to identify what pathways are still active in resistant tumors. Fortunately, PI3K signaling is still active in tumors resistant to CDK4/6 inhibitors. This makes it a viable pathway to target in tumors that do not respond to the inhibition of the cyclin D–CDK4/6–Rb pathway. Follow-up animal studies showed that alpelisib, a p110α-selective PI3K inhibitor, prevented the progression of tumors in ER+ xenografts resistant to CDK4/6 inhibitors [79]. PI3K inhibitors are effective because they reduce levels of cyclin D1, along with other cyclins that work during the G1–S transition. This drug also helps to prevent the phosphorylation of Rb and inhibit the transcription of genes needed to transition to S phase [43]. 

PDK1 inhibitors are in development to overcome resistance by deactivating the PI3K pathway. Two of these drugs, SNS-229 and SNS-510, have been shown to successfully prevent PDK1 phosphorylation and prevent signaling through the PI3K–AKT pathway. This deactivation also correlated with decreased proliferation in various models of hematological cancer, including multiple myeloma, acute myeloid leukemia, diffuse large B-cell lymphoma, and mantle cell lymphoma in vitro. Experiments in xenografts reflect the efficacy of inhibiting PDK1. After 3 weeks of being treated with either PDK1 inhibitor, more than 95% of tumors had notable growth inhibition, and over 50% even experienced regression [80]. Though these experiments were performed in models of hematological cancers, the authors note that the drug also has promise in solid tumors that have overactive PDK1.

Multiple clinical trials utilizing PI3K inhibitors have been initiated. One in particular, the phase II BYLieve study (NCT03056755.), looked at the efficacy of alpelisib, a PI3K inhibitor, with fulvestrant in HR+/HER2-, PIK3CA-mutant advanced breast cancer after patients had previously progressed on a CDK4/6 inhibitor and aromatase inhibitor [81]. These patients were given 300 mg alpelisib per day combined with 500 mg fulvestrant on day 1 and 15 of the first 28-day cycle, followed by 500 mg fulvestrant on the first day of all subsequent cycles. During treatment, the most common grade 3/4 adverse events occurred in 26% of patients and included hyperglycemia and rashes. The primary endpoint, defined as patients who had not yet progressed after 6 months, was met by 50.4% of patients. Furthermore, the average PFS was 7.3 months. This trial also looked at the efficacy of combining alpelisib with letrozole in patients previously treated with a CDK4/6 inhibitor and fulvestrant. Patients in this subset were given 300 mg alpelisib and 2.5 mg letrozole daily. After 6 months, 46.1% of patients had not progressed, and the PFS of this cohort was 5.7 months [82]. Common adverse events were diarrhea, nausea, and decreased appetite, while severe events included hyperglycemia and rashes. Though this clinical trial did not include a control group for comparison of efficacy, it was determined that combination therapy with alpelisib is useful in ER+ breast cancer patients whose tumors progressed while on CDK4/6 inhibitors.

Aside from using PI3K inhibitors to overcome resistance, adding a PI3K inhibitor to CDK4/6 inhibitor treatment is also successful at prolonging initial resistance from developing. Vora et al. [83] utilized xenograft models to show the efficacy of combining a CDK4/6 inhibitor with a PI3K inhibitor. It was determined that these drugs work synergistically to reduce cell viability. The mechanism through which this works is by reducing Rb phosphorylation, which helps to keep Rb functioning as a brake on the cell cycle. As a result, this treatment combination would likely increase the effectiveness of CDK4/6 inhibitors, and potentially circumvent drug resistance, in ER+ patients who have WT Rb.

Furthermore, O’Brien et al. [79] emphasized the potential of including a PI3K inhibitor in the initial treatment regimen for patients with ER+ breast cancer. They used xenograft models of ER+ breast cancer to determine the response to alpelisib if it is administered with current standard of care. Tumor volume decreased the most in xenografts given alpelisib followed by a combination of alpelisib, ribociclib, and fulvestrant than in mice treated with any two of these three or monotherapy. As a result, these authors suggest triple combination therapy of an antiestrogen, CDK4/6 inhibitor, and PI3K inhibitor as the primary intervention for treatment-naive patients.

Moreover, Herrera-Abreu et al. [43] found that when PI3K inhibitors are used in combination with CDK4/6 inhibitors in vitro and in PDX models of ER+ breast cancer, cells undergo apoptosis. When comparing PDX response to combination therapies including fulvestrant, ribociclib, and a PI3K inhibitor (BYL719), the regimen including all three together led to the largest decrease in tumor mass. In fact, after 47 days of treatment, 87% of the tumor had regressed. Based on this, it was determined that a treatment combination including all three is the most effective option, particularly for prolonging the development of resistance. 

### 6.3. mTOR Inhibitors

Inhibiting mTOR has the potential to overcome acquired resistance to CDK4/6 inhibitors. O’Brien et al. [79] show that CDK4/6 inhibitor-resistant xenografts treated with everolimus, an mTOR inhibitor, and fulvestrant have the largest decrease in tumor volume over the course of about 115 days compared to when fulvestrant is paired with either ribociclib or alpelisib. This suggests that using an mTOR inhibitor can potentially reverse resistance to CDK4/6 inhibitors.

Clinical data show that mTOR inhibitors can have a modest effect in overcoming resistance. In a retrospective study analyzing 41 metastatic breast cancer patients who progressed on palbociclib, the outcomes of patients given various doses of everolimus were tracked. While the majority of patients’ cancer progressed, 17.1% of patients had an objective response and clinical benefit [84]. Notably, two patients achieved a prolonged benefit of 13 months and 16 months. While these results are not particularly impressive, they do show that everolimus has potential to provide benefit to some patients.

Another retrospective study to include patients who became resistant to CDK4/6 inhibitors looked at the efficacy of everolimus, but in combination with exemestane, a steroidal aromatase inhibitor. Seventeen total patients were identified to be treated with a CDK4/6 inhibitor prior to everolimus/exemestane combination therapy [85]. Of these, 16 were given palbociclib and one was given ribociclib. While stomatitis was a common side effect, patients did experience a mild clinical benefit. The PFS of patients with CDK4/6 inhibitor resistance was 15.6 months, compared to 11.3 months in patients who did not receive CDK4/6 inhibitors prior to everolimus/exemestane treatment. While this is not a significant difference, it does show that everolimus has some efficacy in patients who previously were treated with a CDK4/6 inhibitor. Based on the mild results, these authors recommend the use of everolimus and exemestane as a second-line therapy to CDK4/6 inhibitor treatment. 

Data obtained in clinical trials also support this notion. A retrospective study compared many different treatment regimens, including everolimus, in patients who progressed after first- or second-line CDK4/6 inhibitor use. Patients were given either everolimus/exemestane, monotherapy with an antiestrogen, or chemotherapy. Of these, the most successful option was the everolimus and exemestane combination, with the median time to treatment failure being 13.2 months, compared to 3.1 months (single-agent hormonal therapy) and 4.1 months (chemotherapy) [86]. This study further supports the use of everolimus and exemestane as the next line of treatment after the development of resistance to CDK4/6 inhibitors.

The recent TRINITI-1 trial (NCT02732119) differs from the others that have explored the use of mTOR inhibitors in ER+ breast cancer patients because patients in this trial were actually treated with ribociclib as part of their regimen even though they had previously progressed on a CDK4/6 inhibitor. The hope behind this is that the other drugs being included will help to overcome the tumor’s resistance mechanism and allow for the CDK4/6 inhibitor to have its efficacy restored. Patients enrolled in this study were split into two groups that each received different dosages of ribociclib, everolimus, and exemestane. Group 1 received 300 mg ribociclib, 2.5 mg everolimus, and 25 mg exemestane daily [87]. Group 2 received 200 mg ribociclib, 5 mg everolimus, and 25 mg exemestane daily. This dosage schedule is particularly noteworthy because patients were given ribociclib continuously over the 28-day cycle without a break. While abnormal, the phase I study that predated this phase II trial recommended this as the optimal schedule when CDK4/6 inhibitors are combined with everolimus and exemestane [88]. While the full results are not yet published, preliminary data show that by week 24, the clinical benefit rate (defined as the number of patients with complete/partial response or stable disease out of the total number of patients enrolled) was 41.1% [87]. It was noted that the most frequently reported adverse events were stomatitis and neutropenia. However, given the percent of patients that still had a response after 6 months, this combination is deemed to be a successful option for advanced ER+ patients who previously have progressed on CDK4/6 inhibitors.

In addition to overcoming resistance, mTOR inhibitors are proposed as a potential way to prolong the development of resistance. In vitro experiments comparing 100 nM vistusertib, an mTOR inhibitor, and 300 nM palbociclib show significant anti-proliferative effects in the cells receiving the drug combination after just five days, compared to cells receiving only one of the drugs. Everolimus also successfully decreases proliferation when used as a monotherapy or in combination with palbociclib in vitro [71].

Using MCF7 xenograft models, Michaloglou et al. [71] also tested the in vivo efficacy of adding an mTOR inhibitor to the current standard of care. Mice treated with vistusertib, palbociclib, and fulvestrant experienced a 110.2% growth inhibition by day 48 of the experiment. Furthermore, the tumors did not begin to grow again over the next 58 days. Overall, this study strongly supports the use of combining CDK4/6 inhibitors and antiestrogens with mTOR inhibitors to delay the onset of ER+ breast cancer resistance. 

### 6.4. FGFR Inhibitors

As previously mentioned, to verify that FGFR1 amplification could be implicated in resistance to CDK4/6 inhibitors, Formisano et al. [66] used lucitanib to reverse resistance in MCF7 and T47D cells. After determining that this was indeed a mechanism cells used to bypass CDK4/6 inhibition, they performed more relevant preclinical studies to determine the effect this drug had on tumor growth in vivo. Athymic mice were implanted with MCF7-FGFR1-overexpressing cells and tumors were allowed to develop. Then, they were either treated with vehicle, fulvestrant, fulvestrant and palbociclib, or fulvestrant, palbociclib, and lucitanib. All three drugs in combination proved to be the most effective at reducing tumor burden. In fact, these tumor volumes were reduced by about 50% after just 3 weeks. Moreover, mice with tumors that did not overexpress FGFR1 did not experience any difference when treated with the triple combination or fulvestrant and palbociclib, which emphasizes that lucitanib is successful at targeting FGFR-overexpressing cells.

Though no trials have evaluated the use of inhibiting FGFR1 in patients resistant to CDK4/6 inhibitors via this mechanism, there is one currently nearing completion (NCT03238196) in metastatic ER+/HER2- patients with amplified FGFR1 [89]. This phase Ib study is determining the safety, tolerability, and efficacy of erdafitinib. Patients were given 4–8 mg of erdafitinib once daily in combination with monthly 500 mg fulvestrant injections and 125 mg palbociclib in the standard of care cycle. Serious adverse events during treatment were rare, but consisted of one each of elevation of transaminases, thromboembolic event, and colitis. Meanwhile, common effects included hyperphosphatemia, dysgeusia, diarrhea, fatigue, and neutropenia. The maximum tolerated dose of erdafitinib was 6 mg, and no interaction was seen with the other two drugs in the trial. The clinical benefit rate was 28% at 6 months post-treatment initiation, and the median PFS was 3 months. Importantly, patients with higher numbers of FGFR1 amplification experienced an improved PFS of 6 months. This modest benefit will be further evaluated in a phase II trial. Overall, since breast cancer patients resistant to CDK4/6 inhibitors can gain resistance due to an FGFR1 amplification, the same therapeutic effect of FGFR1 inhibition will likely be seen if a future study focuses on this specific population.

### 6.5. CDK2 Inhibitors

Due to its involvement in causing resistance to CDK4/6 inhibitors, targeting CDK2 has been proposed as a way for cells to regain sensitivity. This idea was tested in MCF7 cells made resistant to palbociclib. First, CDK2 siRNA was transfected into cells treated with palbociclib to see if CDK2 knockdown allowed cells to regain a response to palbociclib [90]. Indeed, knockdown of CDK2 rendered the previously resistant cells responsive to the CDK4/6 inhibitor. Western blot analysis was then used to verify that CDK2 and CDK4/6 inhibition is synergistic because it leads to a decrease in phospho-C-MYC, which destabilizes C-MYC [90]. When C-MYC is phosphorylated, it is stable and allows cells to escape senescence. These experiments were repeated in xenograft models and achieved the same results. In summation, these experiments show that inhibiting CDK2 can assist cells in overcoming resistance to CDK4/6 inhibitors by forcing them to become senescent.

Multiple small molecule inhibitors of CDK2 are currently in development for intended use in patients who have intrinsic or acquired resistance to CDK4/6 inhibitors. In CDK4/6 inhibitor-resistant cells in vitro, the CDK2 inhibitors both decrease proliferation and arrest cells in the G2/M phase [91]. These experiments were also repeated in mouse xenografts to evaluate the efficacy of these drugs in an in vivo model of acquired resistance to CDK4/6 inhibitors. Lim et al. [2] also display that targeting CDK2 with CYC065, a CDK2/9 inhibitor, helps MCF7 cells and MCF7-derived xenografts resistant to CDK4/6 inhibitors and endocrine therapy regain sensitivity. Excitingly, pharmacological CDK2 inhibition is a promising novel way to overcome resistance to CDK4/6 inhibitors.

### 6.6. Chemotherapy

Unfortunately, tumors that develop resistance due to a loss of Rb are not able to have that alteration directly targeted. Experiments to determine if the loss of this tumor suppressor is associated with a more favorable response to chemotherapy were used to see if this is a viable alternative treatment option. Using gene expression datasets from breast cancer patients, researchers aimed to determine if patients with decreased Rb signaling responded better to chemotherapy than patients with Rb intact [92]. It was determined that breast cancer patients with mutated Rb given three different regimens of chemotherapy (5-flurouracil/adriamycin/cytoxan, taxane/adriamycin/cytoxan, and taxane/5-flurouracil/adriamycin/cytoxan) generally had a complete response to treatment. Fortunately, however, these tumors are typically sensitive to chemotherapy because mutations in Rb actually sensitize cells to DNA damaging agents. This analysis was also used after assessing Rb levels with immunohistochemistry (IHC) on samples from pretreatment biopsies, and determined the same result. Importantly, IHC analysis on biopsy samples can be easily used in a clinical setting prior to the administration of treatment to evaluate if a patient will have a favorable response to chemotherapy.

While chemotherapy is often used in the clinic after progression on CDK4/6 inhibitors, almost no trials have really evaluated if this is a particularly effective strategy. To gather data on this, Rossi et al. [93] analyzed a subset of patient outcomes from the previously mentioned TREnd trial. Once progression occurred and their participation in the trial was over, patients who received chemotherapy or endocrine therapy were followed-up on for this retrospective study. The median time-to-treatment failure of patients who received chemotherapy was 4.6 months, compared to 3.7 months in patients who received endocrine therapy [93]. While this time is minimal, it does suggest that patients who progress on CDK4/6 inhibitors do achieve a small benefit from undergoing chemotherapy. 

The use of chemotherapy in combination with CDK4/6 inhibitors to increase their efficacy as a first-line option is currently being studied. If the two drugs behave synergistically in patients, it is hoped that chemotherapy can help to prolong, or even prevent, resistance to CDK4/6 inhibitors from arising. One phase I trial (NCT01320592) was the first to look at the use of palbociclib in combination with paclitaxel, a chemotherapeutic agent, to see if the two drugs work well together and are tolerated in patients with advanced breast cancer [94]. Fifty milligrams of palbociclib was administered 5 days at a time, followed by a 2-day break, for three weeks in a 28-day cycle. Patients were given paclitaxel once per week on the day before beginning the palbociclib regimen, and on day 22 during the week they did not receive palbociclib. This schedule continued for three months. Afterward, it remained the same except paclitaxel was no longer given during the week of the palbociclib break. Patients who stopped having a response after six cycles were given the opportunity to increase their palbociclib dose to the next highest option: 75 mg, 100 mg, or 125 mg. Importantly, the two drugs are not able to be administered on the same day because preclinical studies show that they actually act antagonistically when taken together [94]. Throughout the course of the study, grade 3/4 neutropenia occurred in many patients and led to some needing reduced doses of palbociclib. However, adverse events were not more common or severe than when either of the drugs is taken alone. The efficacy of treatment was determined by both the response rate and PFS and varied based on palbociclib dosage. Response rates were 50%, 44%, 50%, and 33.3% for the 50 mg, 75 mg, 100 mg, and 125 mg groups. The median PFS for each group in increasing order was 105, 812, 234, and 209 days. Overall, this study demonstrated that combining CDK4/6 inhibitors with chemotherapy is a safe and effective combination. Future studies need to compare the PFS and response rates in patients given this combination to patients given CDK4/6 inhibitor monotherapy to evaluate if it is a viable method of prolonging resistance to CDK4/6 inhibitors in breast cancer patients.

### 6.7. Autophagy Inhibitors

Autophagy inhibitors are a promising treatment option after resistance to CDK4/6 inhibitors arises. Inhibiting autophagy by shRNA downregulation of two autophagy genes, Atg-5 and Beclin-1, sensitizes ER+ breast cancer cells to palbociclib and results in irreversible cell cycle arrest and growth inhibition [60]. These same results were also observed in cells and xenografts treated with both palbociclib and hydroxychloroquine, an autophagy inhibitor. One possible mechanism of how these drugs synergize is that autophagy typically degrades reactive oxygen species (ROS). Autophagy inhibitors cause ROS to be present at higher levels, which induces senescence and apoptosis [60].

As shown in triple-negative breast cancer resistant to anthracyclines, autophagy inhibitors successfully supplement anthracyclines to decrease proliferation and viability. The induction of cell death via apoptosis is another important mechanism by which autophagy inhibitors work. Molecular knockdown of autophagy gene products Autophagy Related 5 (ATG5) and Autophagy Related 7 (ATG7) prior to a caspase assay displayed that a reduction in autophagy is associated with an increase in caspase-3 and caspase-7 in resistant cells. The induction of caspase-dependent cell death by the inhibition of autophagy is one reason to target autophagy in cells that rely on it to cope with drug treatment [59]. Since autophagy is a known mechanism of ER+ tumor resistance to CDK4/6 inhibitors, future studies should focus on repeating this experiment with models of this subtype of breast cancer to confirm that this mechanism of overcoming resistance is also valid in the ER+ population.

There is currently a clinical trial underway (NCT04841148) that is evaluating the efficacy of hydroxychloroquine in combination with palbociclib at preventing recurrence in ER+ breast cancer patients with bone metastases. Since autophagy is a mechanism that allows breast cancer cells to become dormant and eventually re-form into a tumor, it is important to test the inhibition of autophagy in a subset of patients who are likely to experience a recurrence. One arm will receive 600 mg hydroxychloroquine twice daily. The other arm will receive 600 mg hydroxychloroquine twice daily and one regimen of palbociclib: either 125 mg daily for 21 days with 7 days off, or 75 mg daily for 28 days [95]. While this trial focuses on metastatic patients, the results will shed light on the potential of combination therapy with an autophagy inhibitor and CDK4/6 inhibitor to overcome resistance in non-metastatic patients as well. If this trial finds that palbociclib and hydroxychloroquine are synergistic and significantly increase recurrence-free survival in metastatic patients, a similar future trial should look at the use of hydroxychloroquine in patients who developed prior resistance to CDK4/6 inhibitors.

### 6.8. eIF4A Inhibitors

The eukaryotic initiation factor 4A (eIF4A) is a translation factor involved in the rate-limiting 5’ remodeling and 40S ribosomal subunit binding step of translation [40]. There are two potent eIF4A inhibitors that are currently available, silvestrol and CR-1-31-B. These cause eIF4A to essentially become stuck to the 5’ leader sequence of an mRNA, preventing the initiation of translation. They also prevent the eIF4A subunit from joining with eIF4F, further inhibiting translation.

The use of eIF4A inhibitors can indirectly affect many different mechanisms of resistance to CDK4/6 inhibitors in the cell. A low dose (3.2 nmol/L) of eIF4A inhibitors in MCF7 and T47D cells prevents the synthesis of cyclin D1, while a high dose (25.6 nmol/L) inhibits cyclin E formation, both of which when upregulated can cause CDK4/6 inhibitor resistance [40]. The combination of palbociclib and CR-1-31-B is synergistic and results in lower levels of cyclin D1 and cyclin E. In turn, the combination causes cell cycle arrest. An in vitro experiment comparing MCF7 parental cells to CDK4/6-resistant derivatives exemplifies that the resistant cells (which also upregulate cyclin D1 and cyclin E1) are more sensitive to this combination therapy. When MCF7 cells are injected into the mammary fat pad of immunodeficient mice, combination therapy with palbociclib and CR-1-31-B significantly decreases tumor growth compared to either monotherapy. Lastly, IHC on these mice revealed that cyclin D1 and cyclin E1 are decreased in the mice treated with the combination therapy, further confirming the mechanism by which eIF4A inhibitors work in this case. Ideally, CR-1-31-B will be tested in xenografts with resistance to CDK4/6 inhibitors in the near future to confirm that these models also experience a therapeutic benefit.

### 6.9. MDM2 Inhibitors

In accordance with the proposed mechanism for how MDM2 can induce breast cancer resistance to CDK4/6 inhibitors, preclinical studies have evaluated the effects of MDM2 inhibitors on the growth of CDK4/6-resistant tumors. MCF7 cells with acquired resistance to both CDK4/6 inhibitors and endocrine therapy regain sensitivity when they are treated with NVP-CGM097, an MDM2 inhibitor [67]. In this model, palbociclib and the MDM2 inhibitor induced cell cycle arrest and caused senescence. Murine xenografts were also used to replicate this work in vivo, and achieved the same results.

Furthermore, one other study has shown that NVP-CGM097 works synergistically with palbociclib, but in models of dedifferentiated liposarcoma. In vitro experiments show that this combination increases apoptosis, while treatment in xenografts shows that the drugs more successfully reduce the tumor growth rate when used in combination [69]. While this work was not performed in breast cancer cells, it is important to know that the induction of apoptosis is a mechanism by which this combination reduces tumor growth, since that is a pathway that is typically deregulated when MDM2 is overactive.

Not many clinical trials have used MDM2 inhibitors in breast cancer patients. However, one phase I/II trial (NCT03566485) is exploring the use of idasanutlin, an MDM2 antagonist, in stage III and IV ER+/HER2− breast cancer patients to determine its utility and safety. In this study, it is being combined with either cobimetinib, an MEK inhibitor, or atezolizumab, a PD-L1 inhibitor [96]. Some of the patients in this trial have progressed while being treated with a CDK4/6 inhibitor; however, that is not mandated in the eligibility criteria for the trial. As such, the results from this trial will give an indication of the potential usefulness of MDM2 inhibition in these specific patients. Once these data are published and it is deemed to be safe, hopefully the drug can be evaluated further in a direct analysis of ER+ patients who are resistant to CDK4/6 inhibitors.

### 6.10. Immunotherapy

Preclinical studies suggest that combining CDK4/6 inhibitors with immunotherapy can help to overcome resistance. Some cancer cells upregulate PD-1, which binds to PD-L1 on a T cell to prevent the T cell from attacking the cancer cell [97]. Anti-PD-1 antibodies bind to PD-1 to prevent this interaction so that T cells can effectively recognize and destroy cancer cells. 

Immunotherapy is newly emerging as a potential way to treat breast cancer. A 2017 paper was the first to display that CDK4/6 inhibitors help to activate the immune system [98]. Mouse models of mammary carcinoma were treated with abemaciclib, and their gene expression was analyzed. Genes involved in antigen processing and presentation were upregulated as a result of the treatment. Furthermore, mice treated with abemaciclib have tumors with reduced expression of Programmed Cell Death Protein 1 (PD-1), Cytotoxic T-lymphocyte-associated Protein 4 (CTLA-4), and other immune-blocking markers, which highlights another anti-tumoral effect of abemaciclib and suggests that immunotherapy might work synergistically with CDK4/6 inhibition. To investigate this, tumor-bearing mice were treated with either abemaciclib or abemaciclib and an anti-PDL1 antibody. The tumors given combination therapy decreased by 70% by day 13 and did not develop resistance through day 35 of treatment. Meanwhile, tumors in mice given abemaciclib monotherapy minimally decreased in size prior to continuing to grow by day 21 of treatment. Excitingly, these results indicate that adding anti-PD-1 immunotherapy to CDK4/6 inhibitors has potential to prolong, or completely stop, resistance to CDK4/6 inhibitors from developing.

One year later, another study built upon this understanding of the synergy between CDK4/6 inhibitors and anti-PD-1 blockade. Mice with existing tumors were treated with trilaciclib, a CDK4/6 inhibitor approved for metastatic non-small cell lung cancer, and tumor volume was subsequently measured [99]. Trilaciclib alone was not sufficient to significantly reduce tumor volume. However, the addition of a PD-1 inhibitor to trilaciclib in mouse-derived spheroids results in widespread cell death. When combining trilaciclib or palbociclib with PD-1 inhibitors in immunocompetent mice, tumors are almost completely ablated. Overall, this study highlights the efficacy of the combinational treatment of CDK4/6 inhibitors and anti-PD-1 antibodies for tumors that are unresponsive to CDK4/6 inhibitors alone.

There is currently an ongoing clinical trial (NCT03147287) that is investigating the effects of combining fulvestrant, palbociclib, and avelumab (another PD-1 inhibitor) in ER+/HER2- patients who had previously developed resistance to palbociclib and endocrine therapy [100]. Patients in this arm of the trial are being compared to patients given either fulvestrant monotherapy or fulvestrant and palbociclib. PFS will be measured over the course of two years as a means by which to compare the efficacy of each treatment regimen. Based on preclinical data, it is expected that the group receiving avelumab will have the longest median PFS. If this is the case, it would strongly support the use of PD-1 inhibitors to help overcome resistance to CDK4/6 inhibitors in the clinic. 

### 6.11. G2/M Checkpoint Inhibition

Since cells that bypass G1 increase reliance on G2, shutting down this checkpoint as well would render the cell unable to repair any DNA damage, thus causing cell death. As previously mentioned, resistant cells upregulate WEE1 and CDK7 to increase activity at the G2/M checkpoint. 

In multiple ER+ breast cancer cell lines, the WEE1 inhibitor AZD1775 (MK1775), which works to decrease WEE1 activity at the G2/M checkpoint, has been shown to increase sensitivity in cells resistant to CDK4/6 inhibitors and cause cell death [72]. Furthermore, proliferation assays on both MCF7 and T47D-resistant cells targeting WEE1 with AZD1775 identified that WEE1 knockdown decreases the proliferation of resistant cells to palbociclib [74]. Fallah et al. [72] also show that AZD1775 decreases proliferation in cells, but does so in cells resistant to all three CDK4/6 inhibitors. Xenograft experiments will follow-up on the in vivo efficacy of AZD1775 in the future.

Aforementioned data by Martin et al. [75] suggest that palbociclib resistance can be overcome by pharmacological targeting of CDK7. THZ1 and its derivative SY-1365 are two drugs that target CDK7 to selectively inhibit its function in G2/M progression [101]. As a result, the use of these drugs arrests cells at G2/M. When comparing the sensitivity of palbociclib-sensitive or palbociclib-resistant ER+ breast cancer cells to these drugs, the resistant cells experience a proliferation decrease similar to the sensitive cells [50]. This is an important indication that resistant cells are responsive to the therapeutic targeting of CDK7. Additionally, combination treatment of SY-1365 with fulvestrant in resistant T47D cells is synergistic, suggesting that this is a potential combination that can be tested in patients to help overcome resistance to CDK4/6 inhibitors. There is currently a clinical trial (NCT03134638) in progress that is testing the efficacy of SY-1365 in combination with fulvestrant in HR+ metastatic patients who previously developed resistance to CDK4/6 inhibitors [101].

## 7. Preventing Resistance to CDK4/6 Inhibitors

Preventing resistance to CDK4/6 inhibitors from arising relies heavily on the development of biomarkers to identify what mechanisms a particular tumor could use to try to circumvent the action of these drugs. Consequently, discovering and screening for these biomarkers can help to inform clinicians as to what treatment regimen would work best based on the individual’s tumor biology. Ideally, this would ensure that patients are not given treatments that will not take effect, and rather steer their doctors to give them a personalized therapy that will target the specific mutations present in their cancer. Altogether, this process is the best imaginable way to prevent resistance from developing in the first place.

Rb and cyclin E status have the potential to be used as determining factors in the response of ER+ breast cancer patients to CDK4/6 inhibitors. The normal G1/S cell cycle checkpoint as indicated by cyclin E negative and Rb positive is a trustworthy biomarker in ER+ patients [60]. Consequently, CCNE1 upregulation and RB1 downregulation are noted to be markers of resistance to palbociclib [46,49]. In accordance with this, high cyclin E expression in ER+ breast cancer patients is indicative of poor prognosis [46]. This same pattern was recognized in a gene expression analysis of 302 patients from the PALOMA-3 trial; patients expressing high levels of CCNE1 did not have a significant response to palbociclib [53,102]. As previously mentioned, Malorni et al. [48] used The Cancer Genome Atlas to find a correlation between Rb inactivity and palbociclib resistance. The predictive model they developed has the capability to identify palbociclib response based on Rb status. Utilizing this model in ER+ breast cancer patients could help to identify which patients are likely to favorably respond to CDK4/6 inhibition and which patients will probably have intrinsic resistance, and therefore should take a different course of treatment.

Breast cancer cells resistant to CDK4/6 inhibitors show upregulation of cystatin S and alpha B-crystallin [61]. Furthermore, these are also upregulated in patients with breast cancer and are correlated with cancer progression. Though their potential role in resistance to CDK4/6 inhibitors has not yet been identified, these can possibly be used as biomarkers to determine which patients will likely not respond to CDK4/6 inhibition.

KRAS is another gene that has potential for use as a biomarker in this case. Serial plasma cell-free DNA genotyping analyses of 106 ER+ breast cancer patients over the course of treatment with palbociclib and fulvestrant were analyzed, and patients who developed KRAS mutations over time became resistant to palbociclib [57]. Consequently, with frequent liquid biopsies, the detection of mutant KRAS in circulating tumor cells can help clinicians determine who will not respond to palbociclib, and likely, the other two CDK4/6 inhibitors. 

FAT1 status is also proposed as a marker of sensitivity to CDK4/6 inhibition. The previously mentioned analysis by Li et al. [64] demonstrated that FAT1 mutation is associated with a drastically worse response to CDK4/6 inhibitors. As a result, the authors of this study strongly support the use of FAT1 as a biomarker for resistance.

CDK4 phosphorylation, specifically at Thr172, is necessary for its activation. It has been hypothesized that without this phosphorylation, the cell would progress through the cell cycle independently of CDK4. Therefore, cells without phospho-CDK4 are expected to be insensitive to CDK4/6 inhibitors. In vitro studies confirmed that sensitivity to palbociclib relies on phospho-CDK4 [103]. Excitingly, a gene expression profile that predicts CDK4 status has since been developed to accurately determine which cells will respond to palbociclib. This qPCR test will be used on data from future clinical trials to further investigate the potential of this tool for use in humans.

## 8. Conclusions and Future Directions

CDK4/6 inhibitors are a relatively new class of drugs that are very effective in improving the response of ER+ breast cancer patients. There are many more aspects about them that need to be further evaluated in the clinic to allow CDK4/6 inhibitors to reach their full potential in breast cancer treatment. Such details that need to be uncovered in clinical trials include their efficacy in early-stage patients, patients with HER2+ disease, and triple-negative breast cancer patients. Furthermore, determining what other drugs synergize with them will help patients to achieve the maximum possible benefit. Trials concerned with all of these aspects are currently underway, and hopefully they will further illuminate the true value of CDK4/6 inhibition in breast cancer therapy.

Though expanding their use would undoubtedly help patients, there also needs to be treatment options for when intrinsic resistance is present or for when acquired resistance eventually develops. Many mechanisms of how breast cancer cells can become resistant to CDK4/6 inhibitors are known at this point, and more are constantly being discovered. In accordance with this, new treatment options need to be created and tested in the clinic to target these complex mechanisms. Ideally, treatments that can be used in combination with CDK4/6 inhibitors to prevent resistance altogether would be the best possible solution. Until this happens, the use of well-established biomarkers prior to administering therapy will help clinicians know which patients will respond favorably to CDK4/6 inhibition and which patients are likely to possess or develop resistance so that personalized, effective treatment can be administered. Altogether, this work will help to solve a challenging clinical problem and drastically improve outcomes for breast cancer patients.

## 9. Materials and Methods

The databases used to gather information for this article include Pubmed.gov and Clinicaltrials.gov. Keywords used to search for references include CDK4/6 inhibitors, CDK4/6 inhibitor resistance, antiestrogens, palbociclib, ribociclib, abemaciclib, ER+ breast cancer, ER+ breast cancer treatment, treating resistance to CDK4/6 inhibitors, chemotherapy for ER+ breast cancer, cell cycle, CDK4/6 inhibitor clinical trials, acquired resistance, and intrinsic resistance.

## Data Availability

Not applicable.
